# The complete mitochondrial genome of *Tuberolachnus salignus* (Gmelin, 1790) (Hemiptera: Aphididae: Lachninae)

**DOI:** 10.1080/23802359.2022.2097024

**Published:** 2022-07-12

**Authors:** Shuang Xu, Xiaolu Zhang, Liyun Jiang, Jing Chen, Gexia Qiao

**Affiliations:** a Key Laboratory of Zoological Systematics and Evolution, Institute of Zoology, Chinese Academy of Sciences, Beijing, China; b College of Life Sciences, University of Chinese Academy of Sciences, Beijing, China

**Keywords:** Mitogenome, giant willow aphid, phylogeny

## Abstract

We sequenced the complete mitochondrial genome of *Tuberolachnus salignus* with a length of 16,868 bp, including 13 protein-coding genes, 22 tRNA genes, 2 rRNA genes, a control region located between *rrnS* and *trnI*, and a repeat region located between *trnS2* and *trnF*. All protein-coding genes are initiated with ATN and terminated with TAA, except the *cox1*, *nad3* and *nad4* with termination of T. The length of the repeat region is 1,407 bp, which repeats 4.04 times with a 305-bp unit. Our phylogenetic analysis showed *T. salignus* is sister to *Stomaphis sinisalicis*.

The aphid *Tuberolachnus salignus* (Gmelin, 1790) has an almost cosmopolitan distribution, which mainly feeds on stems and branches of *Salix* as well as occasionally recorded from *Populus* (Blackman and Eastop 2021), and can build up very large colonies in late summer (Heie 2009). This aphid is very large with 5.0–5.8 mm in length; apterae are mid-brown to dark brown with several rows of black sclerotic patches, have a large dark brown tubercle in the center of abdominal tergites, just in front of the siphunculi which are on large dark cones (Blackman and Eastop 2021). *T. salignus* apparently are anholocyclic everywhere, reproducing exclusively by parthenogenesis; and no sexual morphs are known (Blackman and Eastop 2021). To date, in Lachninae, only one complete mitochondrial genome of *Stomaphis sinisalicis* has been reported (Zhang et al. 2021). Here, we sequenced the complete mitochondrial genome of *T. salignus* on Illumina NovaSeq 6000 platform. Adult apterous viviparous female was used for research, and total genomic DNA was extracted from seven adult aphid individuals. The mitochondrion genes were assembled using SPAdes version 3.15.0 (Bankevich et al. 2012), and annotated using the online MITOS tool (Bernt et al. 2013) to predict protein coding genes, transfer RNA (tRNA) genes, and ribosome RNA (rRNA) genes. The aphid samples were collected in August 2014 from Baishan, Jilin, China (41.959°N, 126.433°E) and deposited in the National Animal Collection Resource Center, Institute of Zoology, Chinese Academy of Sciences, Beijing, China (NZMC No. 32236, Fang Fang, fangfang_bio@126.com; Bin Zhang, zhangbin@imnu.edu.cn).

The complete mitochondrial genome of *T. salignus* is 16,868 bp in size (GenBank accession number OK642815), containing 13 protein-coding genes (PCGs), 22 transfer RNA genes (tRNAs), 2 ribosomal RNA genes (rRNAs), a control region, and a repeat region. The genes are arranged in a different order from the inferred ancestral insect mitochondrial genome (Clary and Wolstenholme 1985). High gene rearrangement has been found in the mitochondrial genome of *T. salignus* between *trnE* and *nad1*, which is similar to *S. sinisalicis* (Zhang et al. 2021). The overall base composition of the whole mitogenome was A (46.1%), T (38.1%), C (10.4%), and G (5.4%), showing strong AT-biased (84.2%).

All PCGs initiated with typical ATN codon and used typical TAG or TAA as the stop codons, except *cox1*, *nad3,* and *nad4* ended with a single T. The tRNAs size ranges from 63 to 73 bp. All tRNAs can be folded into a typical clover-leaf secondary structure except for *trnS1*, which loses the dihydrouridine (DHU) arm. The *rrnL* and *rrnS* genes are 1316 and 772 bp long, with 85.2% and 84.7% A + T content, respectively. The control region is 737 bp in length located between *rrnS* and *trnI*, showing high A + T content (86.2%). The repeat region between *trnS2* and *trnF* is 1407 bp long with an A + T content of 87.0% in the mitogenome of *T. salignus*. A 305-bp repeat unit repeats 4.04 times in this region.

We reconstructed a maximum-likelihood phylogenetic tree with 13 protein-coding genes of *Tuberolachnus salignus* and 30 other aphids including one outgroup species *Adelges tsugae* using RAxML v8.2.10 (Stamatakis 2014). The Lachninae was recovered as monophyletic and formed a basal clade (Figure 1). Besides, *T. salignus* was presented as a sister to *S. sinisalicis* with well-support.

**Figure 1.**
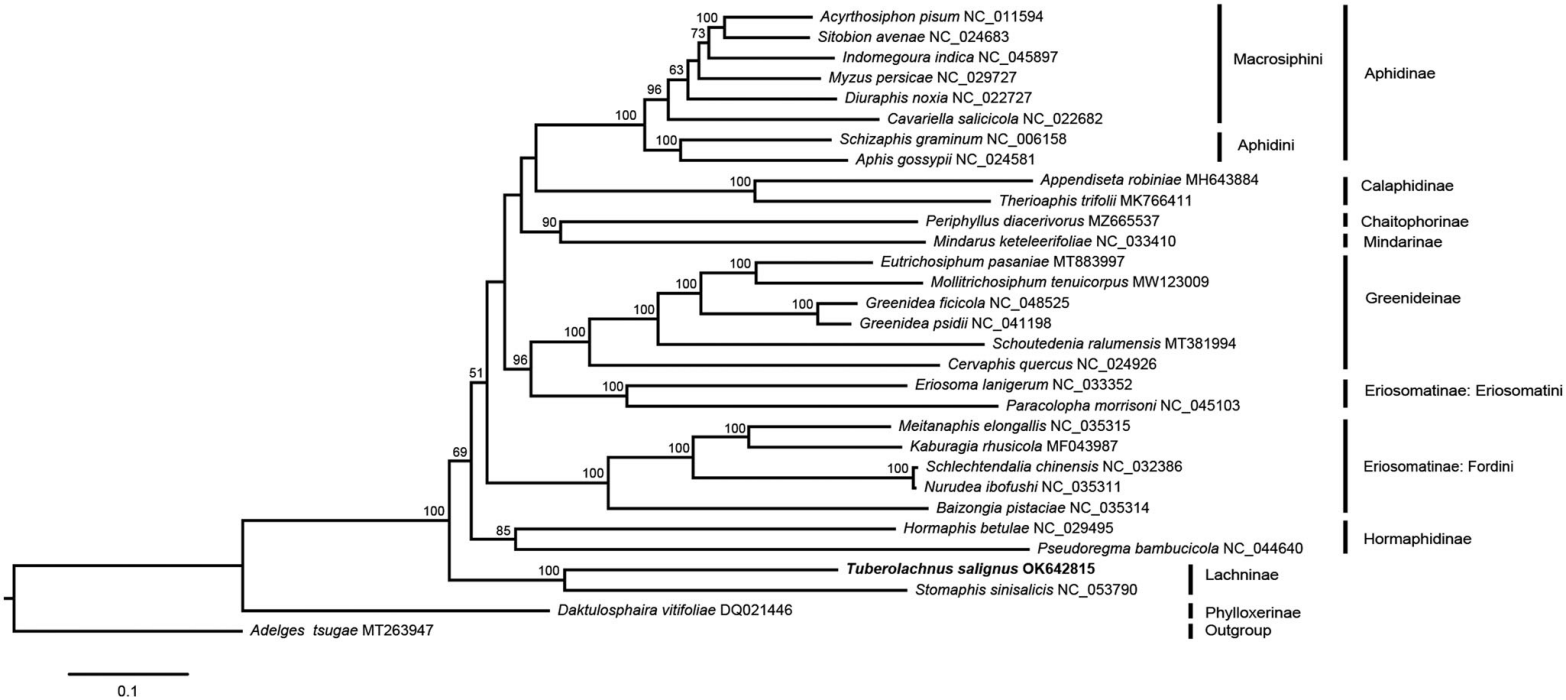
The maximum-likelihood phylogenetic tree inferred from 13 protein-coding genes of *Tuberolachnus salignus* and 30 other aphids. Bootstrap values (>50%) are shown above the branches.

## Author contributions

Gexia Qiao, Jing Chen and Shuang Xu contributed to the conception and design of the study. Shuang Xu and Xiaolu Zhang conducted the analysis and interpretation of the data. Gexia Qiao, Liyun Jiang and Shuang Xu determined the final published version. All authors agree to be accountable for all aspects of the work.

## Ethical approval

*Tuberolachnus salignus* is a common pest on *Salix* in China as well as not recorded in the species list-of-ethics committees for research involving animals of the Institute of Zoology Chinese Academy of Sciences. Therefore, no ethical approval or other relevant permission can be provided for the study.

## Data Availability

The genome sequence data that support the findings of this study are openly available in GenBank of NCBI at https://www.ncbi.nlm.nih.gov under the accession no. OK642815. The associated BioProject, Bio-Sample and SRA numbers are PRJNA773214, SAMN22448656 and SRR16509480, respectively.
